# Automatic sleep spindle detection: benchmarking with fine temporal resolution using open science tools

**DOI:** 10.3389/fnhum.2015.00353

**Published:** 2015-06-24

**Authors:** Christian O'Reilly, Tore Nielsen

**Affiliations:** ^1^MEG Laboratory, McConnell Brain Imaging Centre, Montreal Neurological Institute, McGill UniversityMontreal, QC, Canada; ^2^Dream and Nightmare Laboratory, Center for Advanced Research in Sleep Medicine, Hôpital du Sacré-Coeur de MontréalMontreal, QC, Canada; ^3^Département de Psychiatrie, Université de MontréalMontreal, QC, Canada

**Keywords:** sleep spindles, automatic detection, temporal resolution, reliability, sensitivity, gold standard, assessment

## Abstract

Sleep spindle properties index cognitive faculties such as memory consolidation and diseases such as major depression. For this reason, scoring sleep spindle properties in polysomnographic recordings has become an important activity in both research and clinical settings. The tediousness of this manual task has motivated efforts for its automation. Although some progress has been made, increasing the temporal accuracy of spindle scoring and improving the performance assessment methodology are two aspects needing more attention. In this paper, four open-access automated spindle detectors with fine temporal resolution are proposed and tested against expert scoring of two proprietary and two open-access databases. Results highlight several findings: (1) that expert scoring and polysomnographic databases are important confounders when comparing the performance of spindle detectors tested using different databases or scorings; (2) because spindles are sparse events, specificity estimates are potentially misleading for assessing automated detector performance; (3) reporting the performance of spindle detectors exclusively with sensitivity and specificity estimates, as is often seen in the literature, is insufficient; including sensitivity, precision and a more comprehensive statistic such as Matthew's correlation coefficient, F1-score, or Cohen's κ is necessary for adequate evaluation; (4) reporting statistics for some reasonable range of decision thresholds provides a much more complete and useful benchmarking; (5) performance differences between tested automated detectors were found to be similar to those between available expert scorings; (6) much more development is needed to effectively compare the performance of spindle detectors developed by different research teams. Finally, this work clarifies a long-standing but only seldomly posed question regarding whether expert scoring truly is a reliable gold standard for sleep spindle assessment.

## Introduction

Sleep spindles are bursts of energy in the 11–16 Hz band with a characteristic waning and waxing oscillation pattern of about 0.5 to 2.0-s duration that arises periodically in electrical signals captured from, for example, implanted electrodes, electroencephalography, or magnetoencephalography. This transient waveform is a hallmark of stage 2 (N2) sleep and a biomarker of some diseases (De Gennaro and Ferrara, 2003; Ferrarelli et al., 2010; Wamsley et al., 2012), cognitive faculties (Tamaki et al., 2008; Fogel and Smith, 2011; van der Helm et al., 2011), and even normal aging (Crowley et al., 2002). Thus, an effort to better characterize the properties of sleep spindles is becoming a priority topic for neuroscience and sleep medicine. A necessary step toward this goal is to establish a commonly accepted method for evaluating the performance of automated sleep spindle scoring systems. Some notable efforts have been made in this direction by Devuyst et al. (2011) who proposed a methodology and a publicly available database. However, as will be discussed later, this database is not sufficient in itself to robustly assess the performance of automated detectors and their assessment method does not respond to certain needs of the community studying sleep spindles. One limitation concerns the use of fine temporal resolution scoring for accurately describing the microstructural features of detected spindles.

The present paper contributes to the enterprise of improving automated tools for the scoring of polysomnographic (PSG) microevents like sleep spindles by describing four different, fine temporal resolution detectors. It also provides a thorough assessment of their performance and draws key conclusions about spindle detector performance assessment in general. In next section (Spindle Scoring Evaluation), we present methodological considerations on how to evaluate the performance of spindle scorers, whether human experts or automated detectors. The Methods section describes the algorithms for the four spindle detectors with modifications to increase their temporal resolution. The developed algorithms are made available in the public domain to help improve reproducibility of research, a challenging goal given the wide-spread use of in-house proprietary algorithms. This section also describes four polysomnographic databases used for our investigation. The Results section assesses the performance of the modified detectors using expert scoring as a gold standard. Results are discussed in the Discussion section and suggestions for future development and assessment of automated spindle detectors are proposed in the Conclusion section.

## Spindle scoring evaluation

### Two different applications, two different sets of requirements

There are two very different contexts within which to score spindles and two distinct sets of requirements for assessing their performance. The first context is to identify spindles as a preprocessing step for subsequent scoring of sleep stages. Indeed, according to both AASM (Iber et al., 2007) and Rechtschaffen and Kales (1968) guidelines, the presence of spindles is a key marker of sleep stage N2. In this context, knowing only if a spindle is present in some time window (e.g., the 30-s page used to score a stage) is sufficient. The second context for scoring spindles is to study their properties in relation to other phenomena such as disease symptoms or cognitive faculties. In this context, sleep stages are generally scored manually before automatic spindle detection is attempted; such stage scoring thus constitutes useful *a priori* information for spindle detection. Here, more precise evaluation of spindle characteristics [frequency, root mean square (RMS), amplitude, etc.] are typically of central interest.

Also in this context, timing attributes of sleep spindles, such as their onset, offset, and duration, are of considerable interest and might even be critical in precisely computing more complex characteristics such as variation of the intra-spindle instantaneous frequency or spatial propagation patterns (e.g., O'Reilly and Nielsen, 2014a,b). However, these characteristics are often overlooked when spindle scoring is undertaken for sleep-staging purposes. For example, in the DREAMS database (Devuyst et al., 2011), one of the experts scored all sleep spindles except two as having exactly a 1-s duration. Although acceptable for sleep stage scoring, such detection is suboptimal for a finer characterization of spindle attributes. It also highlights a weakness of human scorers in comparison to automated systems: experts may interpret or apply scoring criteria differently depending on the application to which they think the spindles will be put.

### Fine temporal assessment of spindle scoring

**Figure 1:**
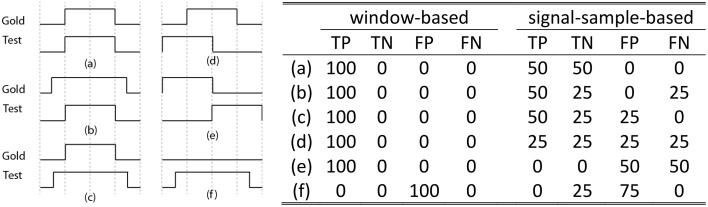
**The left panel shows six common situations [labeled as (a–f)] occurring when comparing the detection of a gold standard scorer (Gold) with another scorer (Test)**. The x-axis on these plots represents time. On the y-axis, a high (low) value indicates the presence (absence) of a spindle. For example, case (a) shows perfect agreement between the gold standard and the tested scorer. Resulting assessments (TN, TP, FP, and FN, in percent) for the proposed signal-sample-based approach and for the window-based method used in Devuyst et al. (2011) are given in rightward panel. Note: The length of the scored signal is taken as being 1 s, such that there is only one decision taken for the window-based method, whereas there are *f_s_* decisions for the signal-sample-based method.

Although the assessment method proposed in Devuyst et al. (2011) might be adequate when spindles are detected for sleep stage scoring, they do not assess sleep spindles with a fine temporal resolution. From this paper, we can only infer that a 1-s scoring window was used for choosing between true positive (TP), false positive (FP), true negative (TN), and false negative (FN) cases as this was not explicitly stated in the methods. A high temporal resolution alternative to this approach would be to consider spindle scoring at a signal-sampling scale (i.e., for a *f_s_* = 256 Hz sampling rate, 256 TP, FP, TN, or FN outcomes are counted per second of recorded signal). As shown in Figure 1, this *signal-sample-based* approach (equivalent to the “by-sample” evaluation in Warby et al., 2014) allows for finer assessment and solves some ambiguities that occur when using a *window-based* approach (as in Devuyst et al., 2011). For example, it is not clear whether condition (e) in Figure 1 should be counted as TP, FP, or FN because the spindles detected by the two scorers are not synchronized. The degree of allowed asynchrony is directly related to the width of the decision window.

### Confusion matrix and related statistics

**Figure 2:**
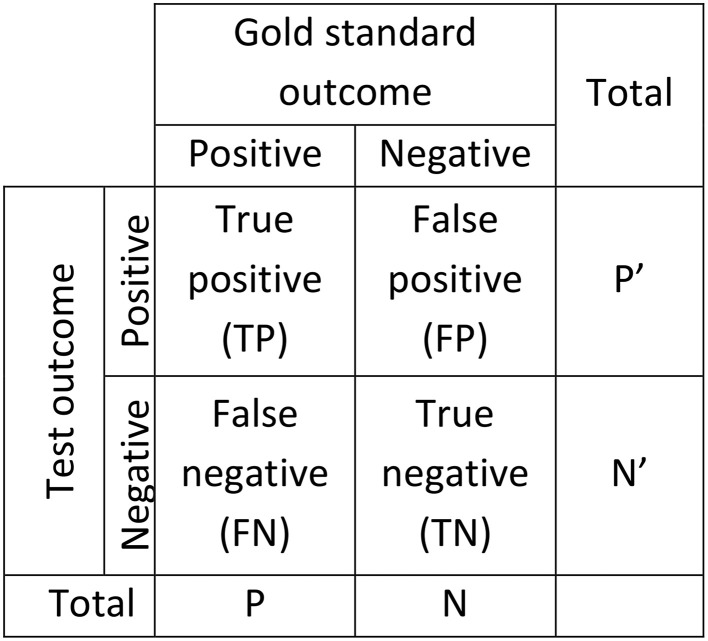
**Confusion matrix used to assess the performance of diagnostic systems**. Two scorings are necessary for this kind of assessment, one considered as giving the true outcome (gold standard) and one for which performance is established as a deviation from the true outcome (Test).

Figure 2 gives the standard confusion matrix used for assessing diagnostic tools. From this matrix, Equations (1)–(3) give the definitions of accuracy, sensitivity (a.k.a. TP rate, recall, hit rate), and specificity (a.k.a. TN rate). These statistics are often used for diagnostic applications in general and for spindle detector assessment in particular.

(1) $$accuracy=\frac{TN+TP}{P+N}$$

(2) $$sensitivity=\frac{TP}{TP+FN}$$

(3) $$specificity=\frac{TN}{FP+TN}$$

(4) $$positive\;predictive\;value=\frac{TP}{FP+TP}$$

(5) $$negative\;predictive\;value=\frac{TN}{FN+TN}$$

Equations (4) and (5) define two other, less frequently used, statistics: the Positive Predictive Value (PPV, a.k.a. precision) and the Negative Predictive Value (NPV). Furthermore, it is noteworthy that the False Discovery Rate (FDR) is linked to the PPV such that *FDR* = 1 − PPV. This is also true for specificity and the False Positive Rate (FPR, a.k.a. fall-out) which are related by *FPR* = 1 − *specificity*.

It should also be noted that accuracy is a measure of agreement between two scorings, and as such, it is independent of which scoring is used as gold standard and which is used as Test. Moreover, sensitivity and PPV are two sides of a coin; sensitivity becomes PPV when the gold standard scorer is interchanged with the Test scorer. This is also true for the relation between NPV and specificity. Thus, by listing the values of these five variables (accuracy, sensitivity, specificity, PPV, NPV) the results of testing a scorer X against a scorer Y are completely known from the outcomes of inverse comparisons.

Devuyst et al. (2011) developed an automatic spindle detector (Au) and compared its performance with the scoring of two human experts (V1 and V2). The average performances of all three, assessed using our signal-sample-based method, are compared in Table 1. The reported statistics are all more conservative than when using the window-based method. For example, the sensitivity of the automated system (Au) is about 65% with a PPV between 30 and 60%, depending on the expert, as compared to a sensitivity of 70% and a PPV of 74% reported in Devuyst et al. (2011).

**Table 1:** **Statistics for the comparison of spindle scorers from (Devuyst et al., 2011)**.

**Gold**	**Test**	**Accuracy**	**Sensitivity**	**PPV**	**Specificity**	**NPV**
V1	Au	95.18	66.13	30.17	96.65	99.02
V1	V2	95.47	56.40	33.81	96.73	98.40
V2	Au	95.90	63.01	58.34	98.03	98.13

*Only time samples from stage 2 sleep are used to calculate these statistics*.

Note that the sleep spindle detection problem shows a large number of negative cases (N) with respect to the number of positive cases (P), e.g., according to the scoring of V1, the ratio between these two variables varies between 30 and 400, depending on the subject. As discussed further in O'Reilly and Nielsen (2013), in these unbalanced situations where *N* » *P*, specificity, NPV, and to a lesser extent, accuracy will always tend to be close to 1. Apparently very good specificity and sensitivity statistics alone may in fact be misleading as they can conceal a very low PPV. Thus, reported outcomes should concentrate on sensitivity and PPV (or equivalently, on the false detection rate) rather than on the typically reported sensitivity-specificity pairs. Furthermore, accuracy should be considered only as a statistic allowing comparison with other detectors and not as a statistic that is sufficient for claiming good performance in its own right.

Also as noted in O'Reilly and Nielsen (2013), these basic statistics are best supplemented with more robust statistics such as Cohen's κ (Cohen, 1960), Matthew's coefficient of correlation (MCC) (Matthews, 1975), or the F-measure—especially in the case of unbalanced datasets. Since none of these measures has yet been established as the standard for spindle scoring, we report results for all three of them.

Cohen's κ coefficient is defined by:

(6) $$\kappa =\frac{accuracy-P_{e}}{1-P_{e}}$$

where *P_e_* is the probability of random agreement (given the bias of both scorers) defined such that:

(7) $$P_{e}=\frac{P^{\prime }P+N^{\prime }N}{{\left(P+N\right)}^{2}}$$

MCC is defined by:

(8) $$\text{MCC=}\frac{\text{TP}*\text{TN}-\text{FP}*\text{FN}}{\sqrt{{\text{P}}^{\prime }*\text{P}*{\text{N}}^{\prime }*\text{N}}}$$

The F-measure is defined by:

(9) $$F_{\beta_{C}}=(1+\beta_{C}^{2})\frac{PPV*sensitivity}{PPV*\beta_{C}^{2}+sensitivity}$$

which is a weighted harmonic mean of PPV and sensitivity with the factor β*C* allows one to put more emphasis on either sensitivity or PPV (Chinchor, 1992). A special case of this measure is the F1-score which weights sensitivity and precision equally. In this case, Equation (9) reduces simply to:

(10) $$F_{1}=\frac{\text{2}TP}{\text{2}TP+FP+FN}$$

### Decision thresholds

Generally, at least at some internal level, automated classifiers produce a decision outcome *X* on a continuous scale, e.g., an estimated probability that a given sample is a positive. In such cases, deciding whether a tested sample should be considered as a positive or a negative applies a decision threshold λ*_d_* such that the sample is considered as positive if *X* ≥ λ*_d_* and as negative if *X* < λ*_d_*. This implies that the statistics (1)–(5) are highly dependent on the value used for λ*_d_*, making the comparison between classifiers difficult if based only on *threshold-dependent* statistics evaluated with some specific decision threshold. To obtain a more complete assessment, it is therefore preferable to evaluate the behavior of these statistics as a function of the decision threshold.

### Threshold-independent analysis

In the context of signal detection, evaluating the performance at a specific decision threshold can be problematic. Indeed, if a first classifier obtains both sensitivity and specificity scores of 0.8 whereas a second classifier obtains scores of 0.75 and 0.85 for the two statistics, it is not clear which classifier should be selected as the best. In such a situation, the choice ultimately depends on the costs associated with FPs and FNs, costs that are often unknown or subject to change over time or situations. Moreover, from these statistics alone it is impossible to know if there is a threshold λ*_d_* such that one classifier will rank higher than the other on both measures simultaneously.

#### Receiver operating characteristic (ROC) curve

ROC curves (see Fawcett, 2006 and Wojtek and David, 2009, for comprehensive overviews) have been proposed precisely to answer this question. They allow assessing classifiers under various operating conditions, i.e., using different values of λ*_d_*.

The ROC curve is a parametric curve in the sensitivity-specificity space parameterized using the decision threshold. That is, every specific λ*_d_* threshold is associated with a (sensitivity, specificity) point on the ROC curve, a random classifier forming a straight diagonal line from coordinates (0, 0) to (1, 1). ROC curves are increasingly used in detection problems including the assessment of spindle detectors.

#### Dealing with asymmetry: the PR curve

Using a measure complementary to the ROC curve such as the Precision-Recall (PR) curve^1^ might also prove useful given the significant asymmetry between the number of negative and positive cases encountered in the spindle detection problem (Davis and Goadrich, 2006; O'Reilly and Nielsen, 2013). In this unbalanced situation, the specificity tends toward very high values for any threshold selected in practical applications because choosing thresholds associated with lower specificity would imply unacceptably low PPV. This results in only a small useful portion of the ROC curve which, therefore, benefits from being complemented with information about the behavior of the PPV statistic. This can be achieved by providing PR curves, which are parametric curves that link the TP rate to PPV, using the decision threshold as parameter. Compared to the ROC curve, the PR curve therefore eschews reliance on specificity and depends upon PPV, a more meaningful statistic for asymmetrical problems.

### Correlations among spindle features

Detectors should also be compared for their ability to extract spindles bearing similar properties. This is probably the most important feature for detectors that are used either for characterizing sleep spindles or for investigating relationships between sleep spindle features (e.g., oscillation frequency, amplitude) and subject characteristics (e.g., age, gender, neuropsychological test scores). To evaluate this aspect of a detector, the average values of spindle features are computed within the spindle sets extracted with respect to both the gold standard and the tested classifier. This is performed separately for every recording condition (recording nights, recording channels). Then correlations between these values are computed across recording conditions using the Spearman's rank correlation coefficient. Such computation is performed for a range of threshold values to evaluate the behavior and the reliability of the detector against threshold variation but also to better assess the optimal operating threshold.

High correlations should be obtained if spindles extracted by the gold standard (e.g., an expert) and a tested classifier are to be considered as assessing the same phenomenon. Indeed, if automated classifiers were to detect many more spindles than an expert (i.e., produce many FPs) but correlations between experts and automated detectors for spindle characteristics were high, we could draw two conclusions. First, that both scorings could be used to obtain similar outcomes and, second, that a higher number of spindles detected by the automated systems would probably not be an indication of FPs from the detector but rather of FNs from the expert.

In this paper, five spindle characteristics are investigated: duration, root-mean-square (RMS) amplitude, frequency slope, mean frequency, and density. Duration is defined as the length of the time window during which a *detection function* is above the decision threshold, as will be discussed more thoroughly when presenting the detectors. The window spanning the duration of the whole sleep spindle is used for RMS computation.

Technical details related to the computation of the frequency slope are described elsewhere (O'Reilly and Nielsen, 2014b). In short, it is calculated as the slope of the linear relationship between the time and the instantaneous average frequency of a spindle oscillation. It assesses the tendency of a spindle oscillating frequency not to be stable in time but to vary more or less linearly. Density is the number of detected spindles per minute. Mean frequency is computed as the average frequency of the fast Fourier transform (FFT) as described in Equation (11).

(11) $$f_{mean}\overset{def}{=}\frac{\int_{10}^{16}f\cdot FFT(f)df}{\int_{10}^{16}FFT(f)df}$$

## Methods

### Databases

Four different PSG databases were used for our investigation. This diversity allowed us to assess the impact of heterogeneous databases on automated scoring and to evaluate the resilience of these detectors when used in different setups. To provide results that are easy to compare with those of other research teams, two of the databases used are open access: the DREAMS database (DDB) (Devuyst, 2013) and the Montreal Archive of Sleep Studies (MASS) (O'Reilly et al., 2014).

DDB contains eight 30 min-long EEG signals recorded on channel CZ-A1, except for two using channel C3-A1. Six recordings were sampled at 200 Hz, one at 100 Hz and one at 50 Hz. Subjects were 4 men and 4 women of about 45 years of age [standard deviation (SD): 8 years] with several different pathologies (dysomnia, restless legs syndrome, insomnia, apnoea/hypopnoea syndrome). Spindles were manually annotated by two experts (V1 and V2; V2 only annotated 6 nights). The authors of this database did not specify which scoring rules experts used for scoring spindles.

As of now, the MASS contains one cohort (C1) of 200 complete-night recordings sampled at 256 Hz and split into five subsets. The second subset (C1/SS2) contains 19 nights from young healthy subjects. For this subset, sleep spindles are scored by two experts (V4 and V5) on N2 epochs and on channel C3 with linked-ear reference. A complete description can be found in O'Reilly et al. (2014). It should be noted that relatively low inter-rater agreement is expected between these two scorers since V4 used traditional AASM scoring rules whereas V5 used an approach similar to (Ray et al., 2010). In this case, both broad-band EEG signals (0.35-35 Hz band) and sigma filtered signals (11-17 Hz band) were used in scoring to facilitate the identification of short duration, small amplitude or obscured (e.g., by delta waves or K-complexes) spindles. Also, no minimal spindle duration was used by V5 and four nights (out of the 19) were not scored due to recordings that were judged to reflect poor quality sleep (e.g., alpha intrusions during N2) or intermittent signal quality/artifact (Fogel, personal communication).

The third database (NDB) is taken from an experiment described in detail in Nielsen et al. (2010). Only the subset of subjects not suffering from nightmares and only the two last recording nights (of a total of three consecutive nights) were used. The NDB subject sample contains 14 men [24.7 ± 5.9 (SD) years old] and 14 women [24.6 ± 6.2 (SD) years old]. Subjects were fitted with 4 referential EEG channels from the international 10–20 electrode placement system (C3, C4, O1, O2); 4 EOG channels; 4 EMG channels; 1 cardiac channel for bipolar ECG; and 1 respiration channel for nasal thermistry. Tracings were scored by trained polysomnographers applying standard criteria and using Harmonie v6.0b software. Sleep spindles were visually scored on either C3 or C4 by an expert (V3) using R&K scoring rules.

The fourth database (SDB) contains 19 complete nights from 10 young and healthy subjects (9 were recorded for two consecutive nights). Subjects were fitted with a complete 10–20 EEG electrode grid; 2 EOG channels; 3 EMG channels; 1 cardiac channel for bipolar ECG. Signals were recorded at 256 Hz using a Grass Model 15 amplifier. A linked-ear reference was used for EEG recording. Tracings were scored by trained polysomnographers applying standard criteria and using Harmonie v6.0b software. Sleep spindles were visually scored on Fz, Cz, and Pz by one of the experts (V4) who also scored the MASS spindles. In this case, spindles were scored when a burst of activity in the 12–16 Hz band was observed for 0.5–2.0 s duration.

In the following, only EEG signals from stage N2 sleep were considered. Table 2 lists the characteristics of these four databases.

**Table 2:** **Specifications of the databases used in our assessment**.

	**DDB**	**NDB**	**MASS (SS2)**	**SDB**
Access	Open	Closed	Open	Closed
Sampling rate	200 Hz in 6 cases; 100 Hz in 1 case; 50 Hz in 1 case	256 Hz	256 Hz	256 Hz
Number of subjects	4 men and 4 women	14 men and 14 women	8 men and 11 women	4 men and 6 women
Number of recordings per subject	1	2	1	2 for 9 subjects; 1 for 1 subject
Age	45.9 ± 8.0	24.7 ± 6.1	23.6 ± 3.7	22.4 ± 2.5
Health	Several different pathologies; see text	Healthy	Healthy	Healthy
Epoch duration	30 s	20 s	20 s	20 s
Stage scoring rules	R&K	R&K	R&K	R&K
Number of epochs per recording	360 ± 0	1483 ± 158.7	1447 ± 126.5	1480 ± 113.4
Number of N2 epochs per recording	215 ± 34.5	582 ± 109.4	689 ± 112.3	833 ± 86.2
Number of scorers	2 (V2 scored only 6 recordings)	1	2 (V5 scored only 15 recordings)	1
Spindle scoring rules	Unknown	R&K	ASSM for V4; see text for V5	0.5–2.0 s duration; 12–16 Hz band
Time-resolved scoring	Yes for V1; no for V2	Yes	Yes	Yes
Scored derivation	CZ-A1 in 6 cases; C3-A1 in 2 cases	C3 or C4 with computed ear-linked reference	C3 with resistor ear-linked reference	Fz, Cz, and Pz with resistor ear-linked reference

*When using the notation X ± Y, X is the mean and Y is the standard deviation*.

### Automatic spindle detection with fine resolution

Many automatic detectors have been developed to address the tedious task of identifying sleep spindles manually (Schimicek et al., 1994; Acır and Güzeliş, 2004; Ventouras et al., 2005; Schonwald et al., 2006; Huupponen et al., 2007; Ahmed et al., 2009; Duman et al., 2009; Devuyst et al., 2011; Babadi et al., 2012). However, no implementation of these detectors has been released to the public domain—see however, other papers of this special issue which propose such open-source detectors (Durka et al., 2015; O'Reilly et al., 2015; Tsanas and Clifford, 2015)—, making it very difficult to reproduce reported results based only on the description of algorithms (Ince et al., 2012).

Moreover, the algorithms of these detectors generally have a coarse temporal resolution of $\pm \frac{W_{l}}{2}$ where *W_l_* is the length of an analysis window typically varying between 200 and 1000 ms. For a better characterization of spindles using fine temporal resolution, we target $\pm \frac{1}{2f_{s}}$. For comparative purposes, we here implement four fine resolution versions of originally coarse resolution detectors described in the literature; these detectors are based on RMS amplitude, sigma index, relative power, and the Teager energy operator. The implemented detectors are part of the Spyndle Python package, a publicly available spindle detection and analysis software toolbox (O'Reilly, 2013c).

**Figure 3:**
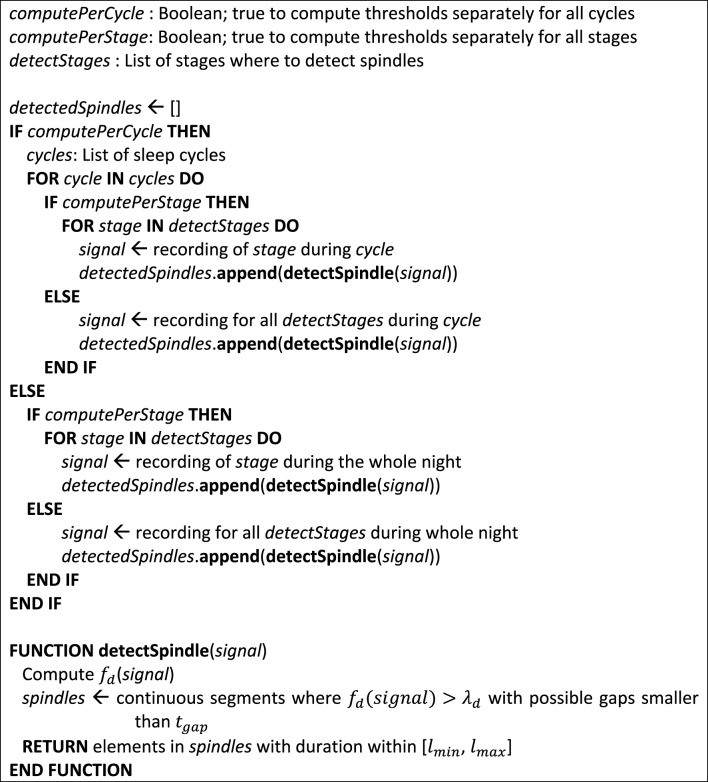
**Pseudo-code for the general architecture of the proposed detectors**. At the end of this algorithm, detected spindles are contained in the detectedSpindles list.

All of the implemented detectors share the same basic structure. They first compute a *detection function f_d_*, i.e., a function whose amplitude varies with the probability of spindle presence. Spindles are detected when *f_d_* exceeds some *effective decision threshold* λ*_d_* for a continuous duration between *l_min_* and *l_max_*. We qualify this threshold as effective to distinguish it from the *common threshold* λ*_c_* (fixed value) from which λ*_d_* is computed (i.e., it can be adaptive or not, depending on the detector). For the investigation reported in this paper *l_min_* was set to 0.5 s—a suggested minimal sleep spindle duration (Iber et al., 2007)—and *l_max_* to 2.0 s to avoid spurious detection of unrealistically long spindles. This upper bound is large enough to capture relevant events considering that spindle duration is generally shorter than 2.0 s; e.g., Silber et al. (2007) reported a 0.5–1.2 s range in young adults. The decision threshold can be either static or vary as a function of the EEG signal assessed for the whole night, the current NREM-REM cycle, or the current stage of the current NREM-REM cycle. For this paper, we used sleep cycles defined as in Aeschbach and Borbely (1993) but other definitions are available as well (e.g., Feinberg and Floyd, 1979; Schulz et al., 1980). We also provide for the possibility of allowing portions of *f_d_* to go below λ*_d_* within the time window spanned by a spindle (i.e., it is a supplementary exception that takes precedence over the *l_min_* criterion) as long as these portions are less than *t_gap_* seconds long^2^. Figure 3 shows the pseudo-code of this general architecture.

**Figure 4:**
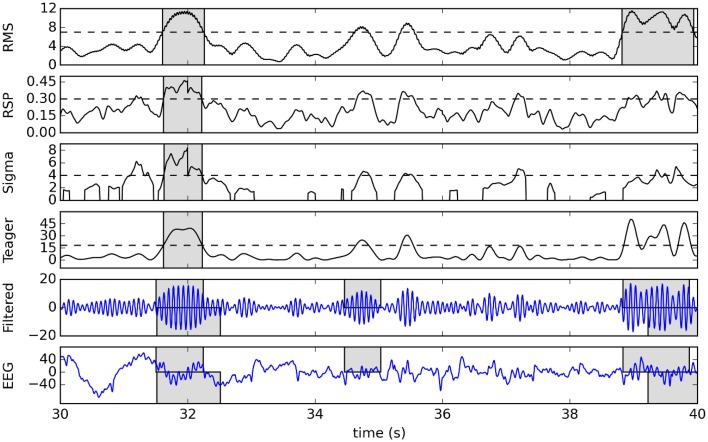
**Example of detection using a 10-s sample from the second subject of DDB**. The original signal and its 11–16 Hz band-passed version are plotted in the two bottom graphs, with gray boxes showing expert scoring (top rectangles for V1, bottom for V2). The four plots in the upper portion of the figure show corresponding detection functions (solid lines), effective thresholds (dashed lines), and detected spindles (gray boxes).

To illustrate this detection process, Figure 4 shows a raw signal from the second subject of DDB and its 11–16 Hz band-passed filtered version as well as the detection function and effective detection thresholds for our four detectors. Detected spindles are indicated by shaded regions.

In the following section, we describe how to obtain the detection function *f_d_* as well as the effective thresholds λ*_d_* for each of our four detectors (see also the Supplementary Materials for related pseudo-codes).

#### RMS amplitude detector

This algorithm is based on a methodology adopted by many researchers in the domain (e.g., Molle et al., 2002; Clemens et al., 2005; Schabus et al., 2007) and initially proposed by Schimicek et al. (1994). Raw EEG signals from each channel are band-pass filtered, rejecting activity outside the spindle band. In our case, we used a 1000th order forward-backward finite impulse response filter with a Hanning window with cut-off frequencies at 11 and 16 Hz. The detection function *f_d_RMS_* is defined as the RMS amplitude of the filtered signal computed within a window of length *W_l_* repeating itself through the entire recording. The value for the effective threshold (λ*_d_RMS_*) is computed as the λ*_c_RMS_* percentile–the 95th percentile is generally used in the literature–of the distribution of the *f_d_RMS_* function. Since the signal amplitude may vary between and within recording nights, this effective threshold is computed separately for every sleep stage of every NREM-REM cycle of a recording.

To increase the time resolution of this method from $\pm \frac{W_{l}}{2}$ to $\pm \frac{1}{2f_{s}}$, the window used to compute the RMS can slide by one sample (maximally overlapped) instead of *W_l_* samples (contiguous) at a time. Using a matrix-based programming language (e.g., Matlab, Python with NumPy), this can be performed efficiently even in night-long signals.

For this paper, a 200-ms averaging window and a *t_gap_* = 0 were used.

#### Sigma index detector

This detector is based on the sigma index (Huupponen et al., 2007). To obtain good time accuracy with an acceptable computational load, we use a time-frequency representation known as the S-transform (ST) (Stockwell et al., 1996) instead of using Fast Fourier Transform (FFT) on contiguous or overlapping windows. The ST is equivalent to a short-time Fourier transform (i.e., a Fourier transform computed over small time periods using a sliding window) with a Gaussian window function whose width varies inversely with the signal frequency. Formally, this transform is expressed as:

(12) $$ST\left(t,f\right)\overset{def}{=}\int_{-\infty }^{+\infty }h(\tau )\frac{\left|f\right|}{\sqrt{\text{2}\pi }}e^{-\frac{{\left(t\text{-}\tau \right)}^{2}f^{2}}{2}}e^{\text{-}i\text{2}\pi f\tau }d\tau$$

with *t* and *f* being transform time and frequency and *h*(t) being the signal to be transformed. For simplicity, we used the discrete version of this transform but a fast version (i.e., similar to what the FFT is to the discrete FT) could also be used if efficiency is an important consideration (Brown et al., 2010).

To minimize processing time, the ST is computed only on the 4–40 Hz band. Since this operation cannot be performed on the whole night at once because of random-access memory limitations and heavy computational overhead^3^, the ST is applied on windows of 4.2 s. Windows are overlapped over 0.2 s and only the 0.1–4.1 range is used to remove artifacts at the temporal borders of the computed transform. Once the *ST*(t, f) array is obtained from the EEG signal, we determine the value *max(t)* = *max_f_spin__* (*ST(t, f_spin_)*) where *f_spin_* = [11, 16] Hz is the frequency range for spindle detection. In other words, *max(t)* is the maximal energy along the frequency axis at a given time t, in the sigma band. We then determine the detection function as the sigma index.

(13) $$f_{\text{d}\_\text{SIGMA}}\left(t\right)=\;\{\begin{array}{cc} 0 & \underset{f_{\alpha }}{ifmax}\left(ST\left(t,\;f_{\alpha }\right)\right)>max\left(t\right)\\ \frac{\text{2}*max\left(t\right)}{m_{l}\left(t\right)\text{+}m_{h}\left(t\right)} & else \end{array}$$

with *m_l_ (t)* = *mean(ST(t, f_l_))*, *m_h_ (t)* = *mean(ST(t, f_h_))*, f_l_ is the 4–10 Hz band, *f_h_* is the 20–40 Hz band, and *f*_α_ is 7.5–10 Hz band. That is, for each time t, the sigma index is the maximal energy in the spindle band normalized by the average between the energy values in the *f_l_* and *f_h_* bands to control for wide band artifacts such as those caused by muscular activity. Moreover, this index is completed by an alpha rejection step which states that the value of the sigma index is canceled out if the maximal energy in the alpha band *f*_α_ is larger than the maximal energy in the sigma band.

Although computed using different signal processing algorithms, the sigma index used here follows the definition proposed in Huupponen et al. (2007). These authors suggest applying a threshold *f_d_SIGMA_(t)* > λ*_d_SIGMA_* with λ*_d_SIGMA_* = λ*_c_SIGMA_* = 4.5. Note that there is no difference between the effective and the common threshold in this case, the effective threshold being taken as a fixed value. We further used *t_gap_* = 0.1.

#### Relative spindle power detector

Following the ideas proposed in Devuyst et al. (2011), we implemented a detection function based on the relative spindle power (RSP).

(14) $$f_{d\_RSP}\left(t\right)=\frac{\int_{11}^{16}ST(t,\;f)df}{\int_{\text{0}.\text{5}}^{40}ST(t,\;f)df}.$$

That is, it represents the instantaneous ratio of the power of the EEG signal in the 11–16 Hz band divided by its power in the 0.5–40 Hz band. Power computation is performed using the S-transform as described in the previous section.

The implementation details for this detector are exactly the same as for the detector based on the sigma index, except that *f_d_SIGMA_ (t)* is changed to *f_d_RSP_ (t)* and an adequate threshold is applied (λ*_d_RSP_* = λ*_c_RSP_* = 0.22 was proposed in Devuyst et al., 2011). We further used *t_gap_* = 0.

#### Teager detector

Based on Ahmed et al. (2009) and Duman et al. (2009), we used the Teager energy operator as another detection function. This operator is defined as:

(15) $$f_{d\_TEAGER}=h^{2}\left(n\right)\text{-}h\left(n-1\right)h\left(n+1\right)$$

where *h*(n) is the digital signal (e.g., the EEG time series in our case) which is transformed into the detection function *f_d_TEAGER_* by the right-hand side of the equation and n is the (discrete) time variable. Duman et al. (2009) propose a decision threshold at λ*_c_TEAGER_* = 60% of the average amplitude (i.e., λ*_d_TEAGER_* = λ_c_TEAGER_^*^ $\bar{f_{d\_TEAGER}}$ where $\bar{f_{d\_TEAGER}}$ is the mean value of *f_d_TEAGER_*). We further used *t_gap_* = 0.

### Scripting

For transparency and better reproducibility of these results, Python scripts used to generate the results presented are provided in the *examples* repertory of the Spyndle package version 0.4.0 available at https://bitbucket.org/christian_oreilly/spyndle.

### Artifacts

No artifact rejection was performed prior to spindle detection. Some detection functions were designed to reject artifacts, e.g., the sigma-index which is designed to reject alpha band activity and muscular artifact. We wanted to test these detectors in the worst conditions to determine their resilience even in the presence of artifacts.

## Results

**Figure 5:**
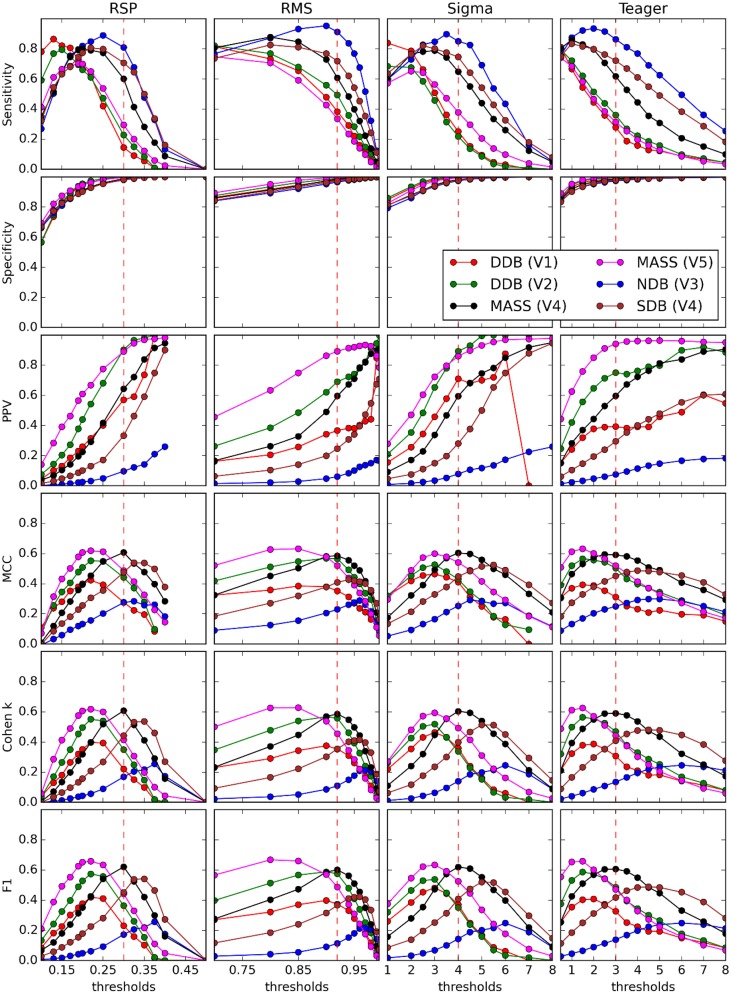
**Performance of four detectors (columns) compared against five experts (V1–V5) scoring four databases (DDB, NDB, MASS, SDB) (see legend on graphs) for six threshold-dependent statistics (rows)**. Vertical dashed red lines show optimal thresholds, as reported in Table 3.

Five analyses performed in this study are described in detail in the next sections. The first compares the detectors against each expert scorer using threshold-dependent statistics computed for a range of decision threshold values (see Table 3 for actual ranges). The second analysis is similar but compares correlations between pairs of detectors/experts for average values of spindle characteristics. The third analysis presents ROC and PR curves for the different detectors using expert scoring as a gold standard. The fourth analysis assesses threshold-dependent statistics for detectors operating with common thresholds judged to be optimal according to our investigations (see Table 3 for corresponding values). These thresholds are subjective choices made by visual inspection following a thorough assessment and motivated by the fact that they balance performance estimates (i.e., attempt to maximize the MCC, F1 and the Cohen κ; see Figure 5) across the expert scorings^4^.

**Table 3:** **Definition for effective thresholds λ*_d_*; tested variation ranges, optimal values according to our investigations, and previously suggested values in the literature for common thresholds λ*_c_***.

**Detector**	**Threshold definition for λ*_d_***	**Tested range for λ*_c_***	**Optimal value for λ*_c_***	**Suggested in literature for λ*_c_***
RSP	λ*_c_RSP_*	[0.1, 0.5]	0.3	0.22
RMS	*percentile*(λ*_c_*, *f_d_RMS__*)	[0.7, 0.995]	0.92	0.95
Sigma	λ*_c_SIGMA_*	[1.0, 8.0]	4.0	4.5
Teager	λ_c_TEAGER_^*^$\bar{f_{d\_TEAGER}}$	[0.5, 8.0]	3.0	0.6

*All effective decision thresholds are applied directly to the corresponding detection functions f_d_. The percentile(p, s) function computes the percentile p of the distribution of a signal s. $\bar{f_{d\_TEAGER}}$ stands for the average value of f_d_TEAGER_*.

A final section presents comparative processing times for the four proposed detectors.

### Comparative performances for threshold-dependent statistics

Figure 5 shows results obtained for threshold-dependent statistics using large ranges of decision thresholds for testing against each expert scoring. Whereas simpler statistics generally monotonically increase (specificity and PPV) or decrease (sensitivity) with respect to the decision threshold, more complete statistics (e.g., Cohen K, F1, and MCC) are low for extreme thresholds and maximal for intermediate values, better capturing the tradeoff between low FPs and FNs.

### Reliability of spindle characteristics

**Figure 6:**
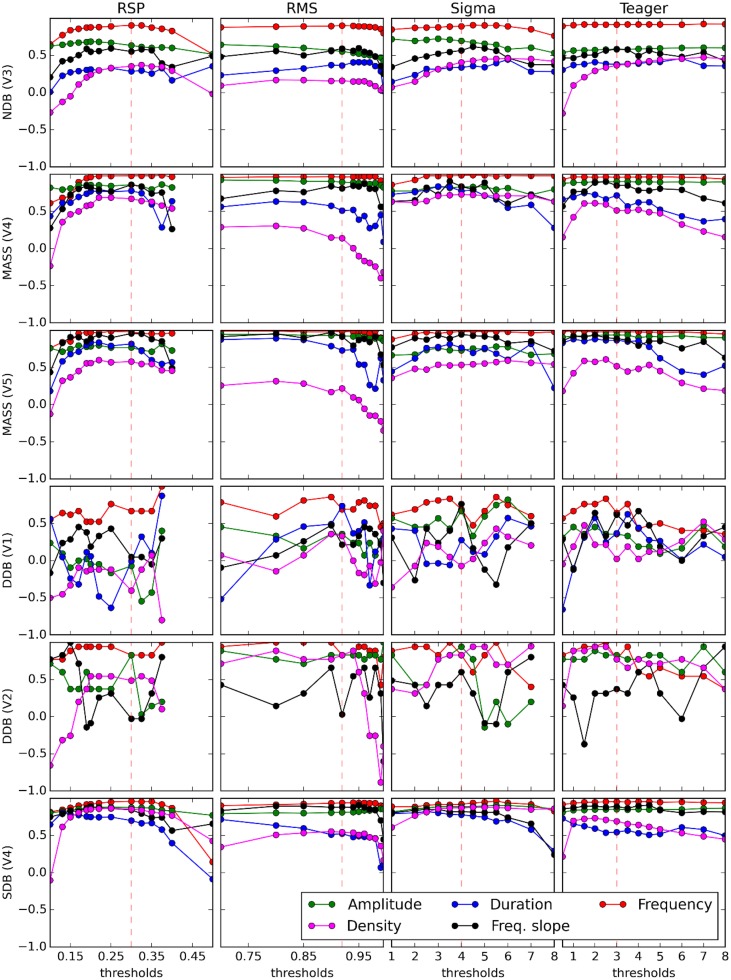
**Curves display variation of the Spearman coefficient of correlation between the median value of spindle features (see legend on graphs) computed from an expert scorer (rows) and an automated detector (columns)**. Decision thresholds are varied in graph abscises. Vertical dashed red lines show optimal thresholds, as reported in Table 3.

Results from previous sections show the extent of the agreement between automated detectors and experts. However, for investigating relationships between sleep spindle properties and subject characteristics it is important to know to what extent the latter relationships are affected by these partial agreements. In other words, we want to verify if these correlations can be reliably assessed regardless of the specific expert or detector used to score spindles. To assess this, the median values of some sleep spindle characteristics (RMS amplitude, density, duration, oscillation mean frequency, instantaneous slope of intra-spindle frequency) are computed for each scored channel of each recorded night. These sets of median values are then compared between pairs of detectors/experts using Spearman correlations. Such computation is performed again for a large range of detection thresholds as shown in Figure 6. In this figure, correlations for V2's estimates of duration are not reported because this expert did not score spindle duration (i.e., every spindle was noted as having a 1-s duration, except for two spindles of 0.49 and 0.5 s).

**Figure 7:**
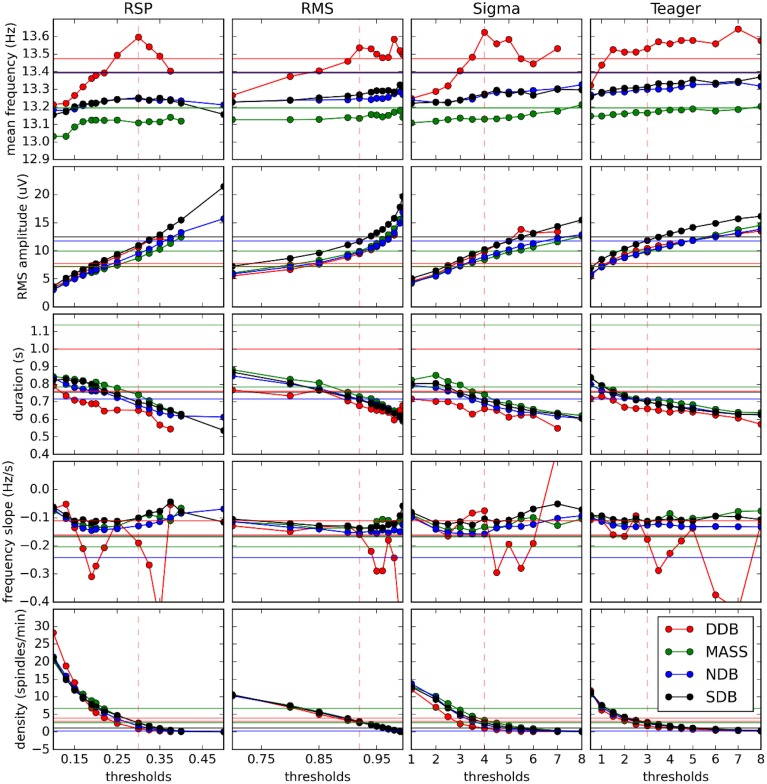
**Variation of spindle characteristics as a function of decision threshold for each of the four automatic detectors**. Straight horizontal lines show values for expert scoring. Vertical dashed red lines show optimal thresholds as reported in Table 3.

Figure 6 shows how spindle characteristics correlate between experts and automatic detectors but do not allow evaluation of whether there is any offset between the different scorings. Presence of such offsets can be assessed in Figure 7 which shows actual spindle characteristic values.

### ROC and PR curves

**Figure 8:**
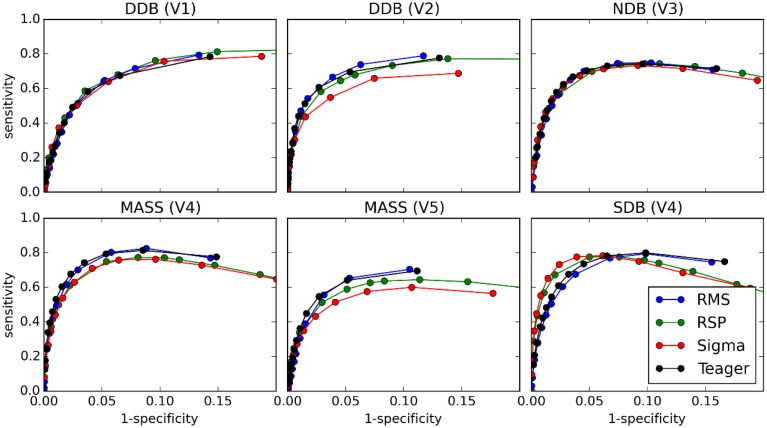
**ROC curves for comparisons between the four classifiers (tests) and scoring by experts (gold standard)**.

**Figure 9:**
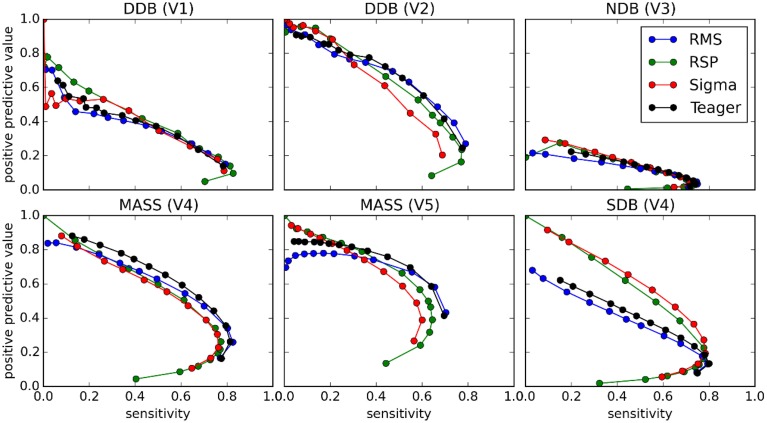
**PR curves for comparisons between the four classifiers (tests) and scoring by experts (gold standard)**.

Figures 8 and 9 show the ROC and PR curves, respectively, for each of the four classifiers. Given the asymmetry of the spindle detection problem, the portion of the ROC curve with specificity less than 0.8 is of no interest since this portion corresponds to useless operating conditions with PPV below 0.2 (this can be observed by comparing specificity and PPV graphs in Figure 5). Thus, ROC graphs have been truncated to focus on the most informative parts.

As can be seen, PR and ROC curves do not increase monotonically, as is generally expected for such curves. This is a consequence of setting an upper limit on spindle duration. Indeed, with such a limit, using lower thresholds causes an increase in sensitivity up to a certain limit, after which excessively long spindles occur and are rejected, lowering the specificity.

### Threshold-dependent statistics at optimal decision threshold

**Figure 10:**
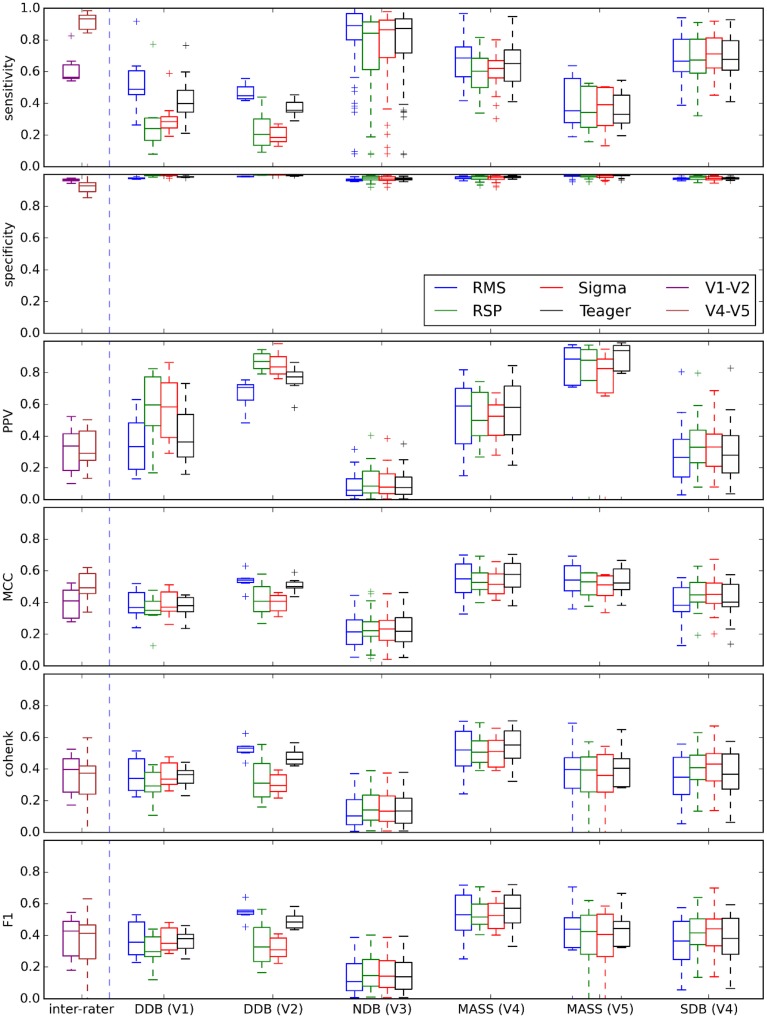
**Box plots summarizing the distribution of threshold-dependent statistics for expert/detector comparisons using decision thresholds judged optimal as reported in Table 3**. Results for each detector are color coded (see the legend). Note that the first two boxes (in purple and brown) at the left of each vertical dashed line show the distributions of statistics comparing experts V1 and V2 (DDB) and V4 and V5 (MASS).

Figure 10 shows performances that can be expected when comparing each expert scoring to the different detectors using optimal decision thresholds as specified in Table 3. Accordingly, these plots would change for a different choice of threshold. Each box represents the distribution of the median value of a given statistic (e.g., specificity) across recording conditions (recording nights, EEG derivations) for a specific expert's scoring [e.g., DDB (V1)] and a specific detector (e.g., RMS).

### Processing time

Computations were performed on Intel Core i7-3970X processors @ 3500 GHZ, using 32 GB of RAM memory (DDR3 @ 800 Hz), running a 64-bit Windows 7 operating system. Since this system has 12 cores and spindle detectors run in single threads, the detection of spindles for all nights, with all 4 detectors, at all threshold values—i.e., detection of spindles for 4488 whole-nights and 408 30-min long signals—was automated and run in 11 parallel detection processes using BlockWork (O'Reilly, 2013b) and EEG Analyzer (O'Reilly, 2013a).

**Figure 11:**
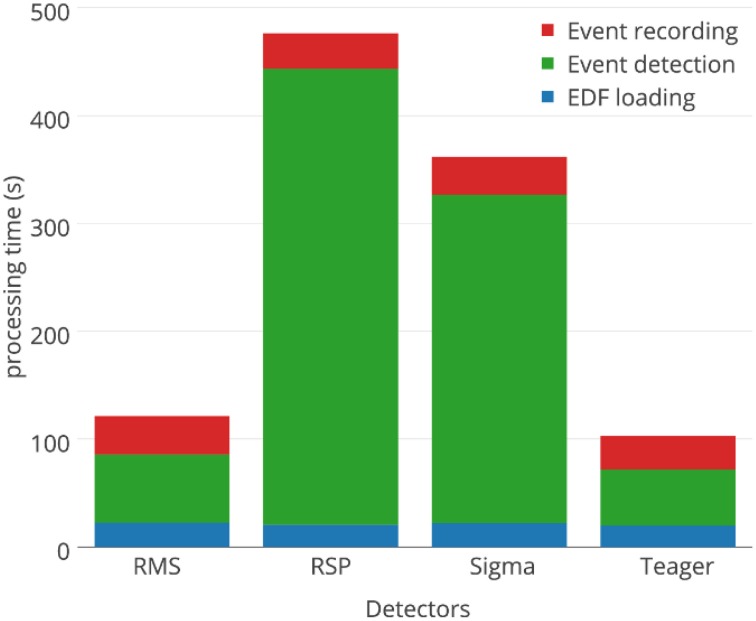
**Comparison of processing times for the four automated spindle detectors**.

Aside from detection performances, processing time required by the detectors is sometimes an important practical constraint. For example, our assessment would have taken about half a CPU-years if spindle detection for a whole-night of EEG signal took 1 h to complete. Fortunately, the proposed detectors are substantially faster. Figure 11 compares the average processing time for each detector, with durations assessed on the MASS nights. Most of the computation time required for spindle detection is associated with three distinct tasks: loading the signals in memory (blue), detecting the spindle (green), and saving the annotations on hard drive (red). As would be expected, only the event detection is significantly affected by the choice of detector. There is about one order of magnitude between the processing time requirements for event detection of the fastest (Teager; 32 s) and slowest (RSP; 402 s) detectors.

## Discussion

### Comparative performance assessment for spindle detectors

As discussed in the Spindle Scoring Evaluation section, the most interesting characteristics for threshold-dependent evaluation of sleep spindle detectors are sensitivity and PPV (precision) as well as more complete statistics such as Cohen's κ, F1-score, and MCC. Specificity is of low interest since the relative scarcity of spindles in sleep EEG forces it to take high values for any reasonable PPV. This is exemplified in Figures 5 and 10. In fact, specificity values can be considered misleading in that they give the false impression that a detector has good performance even if it is not necessarily the case. In light of this, it appears prudent to report PPV or FDR instead of specificity as a measure of a detector's ability to reject FPs.

It is, however, obvious from Figure 9 that the impact of the choice of an expert/database combination has even more influence on PPV than the choice of a detector. This highlights the fact that PPV is directly related to how conservative the expert is when detecting spindles (i.e., the extent to which an expert systematically scores fewer spindles per night than do other experts; see also spread of the optima for MCC, Cohen's κ, and F1-score in Figure 5 which depicts the same phenomenon). It suggests that PPV is more indicative of the relative importance of FNs from the expert part than FPs from the detector part. In this context, it appears ill-advised to compare spindle detectors for which assessments were performed on different databases or different expert scorings. Indeed, the expert scoring and database are two important confounding factors that can completely mask true differences in detector performance. The importance of these confounders on PPV is particularly obvious, but is clearly also true of the other performance statistics (sensitivity, MCC, F1-score, Cohen κ) as can be seen in Figure 10. Fortunately, open-access databases that can be used for comparative purposes are starting to become available. We hope that the present results will incite researchers to propose additional open-access databases or to contribute to existing ones.

Choosing the best decision threshold is rather difficult and almost impossible to do objectively using sensitivity and PPV curves (Figure 5), ROC curves (Figure 8), or PR curves (Figure 9). Such a choice requires estimation of the costs associated with both FP and FN errors. Since these costs are difficult to evaluate and can vary depending on context, MCC, F1-score, and Cohen's κ provide attractive alternatives. These three statistics give very similar assessments with clearly identifiable maxima close to FN/FP tradeoffs that are generally adopted in the literature. Since correlation coefficients are well understood by the general scientific community whereas use of Cohen's κ is restricted more to the field of psychology, MCC might be a good choice of statistics to report. Furthermore, MCC lends itself readily to parametrical statistical analysis since it is related to the χ^2^ distribution (Baldi et al., 2000). The F-measure, on the other hand, has the advantage of explicitly specifying weights on the relative importance of sensitivity versus PPV, whereas the tradeoff is implicit in MCC and Cohen's κ. Similarly, the F1-score implicitly considers these two statistics as being of equal importance, something that might not be true in general. Regardless, no consensus has yet emerged concerning which of these three statistics is best to report, but reporting all three might be preferable when assessing a detector on an open-access database so as to maximize the possibility of comparing detector performances across studies. In any case, at least one such statistic should be reported to provide a more comprehensive view of the detector's performance.

Another important conclusion is that there is an inherent difficulty deciding which automated detector performs best relative to expert scoring using statistics computed at only one specific threshold. Shifts in the decision threshold can produce very different results. Thus, reporting the value of threshold-dependent statistics over some reasonable range of decision thresholds is desirable.

It should also be noted that, because databases and experts constitute two important sources of variability, one should exercise caution in comparing results from studies presenting algorithms that use general classification rules based on heuristics (e.g., the detectors proposed here) with those from studies using detectors that are trained on a database of pre-scored spindles (e.g., Acır and Güzeliş, 2004) unless the training and the testing subsets in the latter are taken from different databases and scored by different experts. Indeed, the maximally attainable performances for heuristic and trained systems are quite different. In the former case, the best performances that can be expected when comparing a detector with different experts are limited by the relatively low average agreement between experts (inter-expert reliability). In the latter case, if scoring from the same expert is used both for training and testing, the maximal performance that the automated detector can attain is only limited by intra-expert reliability.

### Impact of scorers on averaged spindle characteristics

As can be seen in Figure 6, the inter-scorer reliability of spindle characteristics can be loosely ranked, from most to least reliable, as follows: frequency, amplitude, frequency slope, duration, and density. This ordering does not seem to be affected much by the choice of detector. It seems, however, that all curves can be displaced up or down by differences in the quality of the database and the expert scoring. Also, it is perhaps concerning to see that spindle density—the most frequently used spindle characteristic in sleep research—is in fact the least reliably evaluated characteristic. This is not surprising though since density is the only characteristic considered here that is not computed by averaging its value across spindles (i.e., the density is defined directly at the subject level as a count whereas the other characteristics are defined at the level of individual spindles and their value at the level of the subject is obtained by averaging across a large number of spindles). Including, for example, 10% more or fewer events in the averaging process may not cause a large difference for stable characteristics. However, this would cause a rather large error (±10%) for density.

Figure 7 also shows that at optimal thresholds there is generally good agreement between the characteristics of spindles labeled by experts and by detectors, with no large offsets between these two kinds of scorings. In this figure, we see that the frequency slope cannot be reliably evaluated on the DDB. This is likely due to the short duration of the recordings (30 min instead of whole nights) which does not allow for the detection of enough spindles to stabilize computation of the median value. This is most visible for the frequency slope because this measure is harder to estimate reliably on individual spindles than are other properties such as average frequency. Except for this specific case and the results for frequency, detectors tend to agree closely across databases, contrary to the experts. This is consistent with the hypothesis that different experts work with different detection thresholds.

### Choice of an open-access database

Results obtained with DDB have a restricted utility because of severe limitations on the features of this database. For example, the DDB is relatively small, containing only 4 h of recording (8 sequences of 30 min) on one channel. This results in unreliable assessment as can be seen in Figure 6. In contrast, the portion of MASS that was scored for spindles is much larger; about 150 h of recording (19 nights of about 8 h). Another limitation of DDB is in its recording parameters. For example, the EEG of one subject is sampled at 50 Hz, which theoretically allows assessment of frequencies up to 25 Hz without aliasing; however, in practice imperfect filtering produces aliasing even at lower frequencies. Figure 10 also shows generally similar agreement between expert scoring on MASS and on DDB, even if low agreement was expected for MASS given the fact that it was scored by two different teams using two different approaches. Thus, using only the DDB does not appear to be sufficient to provide a robust assessment of spindle detectors and a more complete database such as MASS is preferable for such a purpose.

On the other hand, DDB has the advantage of presenting signals for clinical cases. These can serve as examples or for case studies. Also, DDB has a high value in open-science for fast validation, teaching, and tutorials since it is directly downloadable on the Internet, something not possible for ethical reasons with MASS.

### ROC and PR curves

Results from ROC and PR curves are not conclusive. Detector rankings according to these curves vary from expert to expert. They may, therefore, not constitute the most appropriate tools for assessing spindle detectors. In a related vein, because of the asymmetry of the spindle detection problem, most of the ROC curve is associated with uninteresting operating conditions. Computing the area under this curve hence produces an aggregated measure that is obtained from mostly useless conditions. Therefore, the area under the ROC curve (AUC) does not appear appropriate for assessing the performance of spindle detectors.

### Choosing the best detector

Even with the thorough assessment proposed here, we cannot with good confidence determine the best classifier. Our ability to do so is limited by the lack of a highly reliable gold standard.

Moreover, the required characteristics of a detector may change depending on the desired application. Here is a short list of some of the most important qualities/features that vary with different applications: (1) requirement or not of sleep stage scoring; (2) rejection or not of artifacts; (3) temporal precision of spindle detection; (4) simplicity of the algorithm; (5) efficiency of the code (e.g., code execution time); (6) overall classification performance; (7) reliability of detected spindle characteristics; (8) capacity for extracting spindles that are correlated with other dependent variables (e.g., neurophysiological and neuropsychological variables).

In general, RMS and Teager detectors are good picks for applications requiring simple deployment and rapid processing. The Sigma detector, however, seems more reliable for estimating spindle characteristics when compared against expert scoring. Further, we found that 0.3, 0.92, 4.0, and 3.0 are appropriate values for decision thresholds used with the RSP, RMS, Sigma, and Teager detectors, respectively. These thresholds are close to previously proposed values for Sigma (4.5 vs. 4.0) and RMS (0.95 vs. 0.92). The threshold for the RSP detector is also not too discrepant from previously proposed values (0.22 vs. 0.30). However, for the Teager detector, the previously proposed threshold is five times lower than the one found here (0.6 vs. 3.0). The reason for such a discrepancy between our results and those of Duman et al. (2009) is presently unknown. This detector seems, however, particularly sensible to characteristics of the database. Thus, finding a better approach to adapt the effective decision threshold to the characteristics of individual subjects might help to stabilize the performance of this detector.

Note also that, except for DDB, our assessment was made on young healthy subjects. This is important because sleep spindle properties (e.g., density, frequency, morphology, spatial distribution) vary with age and brain and sleep disorders, such as sleep apnea. These associations have practical implications for using these detectors on clinical datasets. For example, recordings taken from the elderly might need lower decision thresholds to accommodate less pronounced spindles in this population. Precision of the detector would evidently suffer from such an accommodation. Thus, a thorough assessment of the behavior of these detectors is advisable before using them with populations known to have smaller amplitude spindles, more artifacts, or smaller signal-to-noise ratios (SNR).

### The problem of the gold standard

As previously mentioned, our results suggest that a significant proportion of the FPs traditionally attributed to automatic detectors might rather be due to FNs from experts. This raises the question of the adequacy of expert scoring as a gold standard for evaluating spindle detectors. The general reliability of expert scoring can indeed be questioned considering that, in our findings, expert scoring has more influence on automatic detection than does the choice of automated detector. This is further supported by the fact that experts V1–V2 and V4–V5 agree more closely with one another than they do with most of the automated detectors (see Figure 10). These results are in line with reports of a relatively low reliability for expert scoring. For example, F1-scores of 72 ± 7% (Cohen κ: 0.52 ± 0.07) for intra-rater agreement and 61 ± 6% (Cohen κ: 0.52 ± 0.07) for inter-rater agreement have been reported by Wendt et al. (2014).

Our results suggest that there is ample room for improvement of automatic spindle detectors. However, the extent of this improvement is unclear because of low reliability of the gold standard currently available for spindle identification. Without a robust gold standard, results will continue to be limited by average inter-rater agreement. Consensus from a large number of crowd-sourced scoring judges is a possible alternative to expert scoring as a gold standard (Warby et al., 2014), but it remains unproven that common agreement of a large number of low-qualification scorers will provide better detection of atypical, unusual or non-obvious spindles than will experts. Low-qualification scorers will in all likelihood show high reliabilities only on large amplitude spindles with large signal-to-noise ratios. Similarly, it is unclear if consensus scoring of a few experts would, in the long run, be retained as a practical solution. This would require substantial resources and runs counter to the tremendous efforts invested in automation of sleep spindle scoring designed to reduce the burden of manual processing to begin with. It might prove to be a sound approach for scoring only subsets of recordings that can be used for training classifiers to detect the entire database (e.g., O'Reilly et al., 2015). Alternatively, manual validation of automatically scored recordings could prove to be quicker for experts than would be manual scoring of the tracings, and thus would provide a reasonable compromise.

Although automated spindle detectors have been in use for several decades, their development and assessment still require substantial work. As they mature, expert scoring will need to be abandoned in favor of criteria based on construct validation results that reflect the growing capacities of computerized automation and statistical assessment. This task could be facilitated by incorporating correlations between detected spindle characteristics and psychological, physiological and demographic dependent variables. We would expect that spindle features obtained from random detectors would correlate only poorly with such variables, whereas spindle features obtained from detectors tapping genuine neurophysiological phenomena would correlate robustly.

### Limitations

Consensus scoring was not pursued for this study but it clearly warrants consideration in future work. For this analysis, double scoring was only available for two databases. The first (DDB) produced rather unreliable results while the second (MASS) produced low inter-expert agreement. Higher inter-rater agreement in MASS could have been pursued by allowing both experts to consult and align their scorings. We would argue, however, that this is not representative of scoring used in the field. We chose instead to ask experts from two different centers to score these recordings as they would in their research. These low agreements are more representative of the variability in expert scoring that we observe between studies published by different centers than is an artificially increased agreement of experts aligning their scoring through consultation.

It is also noteworthy that no artifact rejection was performed prior to spindle detection. Thus, our results show the relative resilience of these detectors to the presence of artifacts. However, in clinical settings where many artifacts are expected, signals should be adequately preprocessed (i.e., cleaned of artifacts) to ensure robust detection. This is especially true for consistent artifacts that might affect the computation of detection thresholds, e.g., the presence of many high-amplitude arousals or flat segments. Fortunately, the use of percentile statistics in the definition of thresholds should render these thresholds relatively robust compared to thresholds based on, e.g., averages and standard deviations, as long as artifacts introduce only a non-significant amount of activity to the top percentiles of the amplitude distribution.

## Conclusion

As we demonstrate in the present paper, assessing the performance of automated spindle detectors is a complex enterprise. The superiority of a new detector can no longer be supported merely by reporting that threshold-dependent variables such as sensitivity and precision are superior to those of previously published detectors. These basic statistics should be supplemented—at a minimum—by more complete statistics such as MCC. However, because there exist no commonly agreed upon testing conditions (i.e., standard databases, relative positive and negative error costs, etc.) and since these conditions may change with different usage contexts, better estimates of external validity (and, thus, a more general validation) can be obtained by reporting the values of these statistics across a range of decision thresholds. The most useful results are obtained by also providing access to the detector source code such that other research teams may test the detector's performance under different conditions. If authors are not willing to share source code, sharing of at least an executable copy with documentation should be considered.

Aside from the dynamics of spindle detectors themselves, other important topics in detector assessment concern the methodological environment of the evaluation. One key topic is the availability of a validated gold standard against which automatic scoring may be evaluated. Expert scoring has been used *de facto* as a trustworthy gold standard, but this assumption is challenged by our results. Although for the present experts will most certainly keep their gold standard status in spindle detection, the definition of a more reliable and commonly agreed upon standard is urgently needed if progress in the domain is to continue.

A second matter needing attention is the availability of EEG databases. As shown in our results, outcomes from different databases can be quite different depending on the database representativeness (i.e., characteristics of the subject sample), size (i.e., are there enough records to obtain stable averages?), and reliability (appropriate sampling frequency, recording equipment, etc.). The availability of shared databases is critical for the development of new algorithms and the benchmarking of various systems on the same set of biological recordings. Pooling of multiple scorings from experts of different research teams could also help in capturing inter-expert variability when developing classifiers that require training.

Unfortunately, the implementation (both executables and source code) of existing sleep spindle detectors described in the literature are not widely available, making their reproducibility, standardization, and benchmarking difficult to attain. In an effort to stimulate progress in this regard, we provide open source spindle detectors for use by the other researchers working in this area (see the Spyndle package, O'Reilly, 2013c) along with a comprehensive assessment of their performance.

## Funding

Funding provided by Canadian Institutes of Health Research (MOP-115125) and Natural Sciences and Engineering Research Council of Canada (312277) to Tore Nielsen and a postdoctoral fellowship to Christian O'Reilly.

### Conflict of interest statement

The authors declare that the research was conducted in the absence of any commercial or financial relationships that could be construed as a potential conflict of interest.

## Footnotes

^1^Also referred to as Positive Tradeoff (PT) curve in (O'Reilly and Nielsen, 2013).

^2^The *t_gap_* parameter is included as a property of the Python classes implementing the spindle detectors. Thus, its value can be easily changed if needed. *t_gap_* values used for the present investigation are reported below for reproducibility purposes, but the impact of this parameter has not been thoroughly tested yet (i.e., it has been used in an informal, trial-and-error, manual optimization) since testing it systematically would add a factor that would render our analyses prohibitively complex and computationally intensive. It is therefore likely that the *t_gap_* values used in this study are suboptimal.

^3^This is true for the discrete ST. However, since the fast ST can be computed in-place (i.e., without additional attribution of memory), it should be computable on the whole night at once.

^4^We consider these thresholds to be a good tradeoff for most uses. However, depending on the application, one might want to give more weight to sensitivity or to precision. For a specific application, one can choose the operating point that will result in expected performances using Figures 5–7.
