# Supplementary material for: Automatic sleep spindle detection: benchmarking with fine temporal resolution using open science tools
Source: Front Hum Neurosci. 2015 Jun 24;9:353. doi: 10.3389/fnhum.2015.00353 (PMC4478395; doi:10.3389/fnhum.2015.00353)
Supplement: Supplementary file 1 [file DataSheet1.DOCX]

**Appendix**

Figures A-2 to A-5 give the pseudo-code to compute the detection functions of the four proposed detectors. These pseudo-codes rely on some auxiliary functions described in Figures A-1.

| **FUNCTION filter**(*signal*)  **RETURN** filtered version of *signal* using a 1001 tap forward-backward FIR filter with a Hanning window and cutting frequencies at 11 and 16 Hz  **END FUNCTION**  **FUNCTION average**(*signal*)  **RETURN** *signal* averaged across time over 0.2s maximally-overlapped windows  **END FUNCTION**  **FUNCTION** $\boldsymbol{ST}(t, f, signal)$  **RETURN** $\int_{-\infty}^{+\infty} signal(\tau)\frac{\left\vert f \right\vert}{\sqrt{2\pi}}e^{-\frac{\left( t-\tau\right)^{2}f^{2}}{2}}e^{-i2\pi f\tau}d\tau$  **END FUNCTION** |
| --- |

Figure A-1. Pseudo-code for auxiliary functions.

| **FUNCTION** $\boldsymbol{f}_{\boldsymbol{d}\boldsymbol{\_}\boldsymbol{RMS}}$(*signal*)  **RETURN** $\sqrt{\boldsymbol{average}({\boldsymbol{filter}(signal)}^{2})}$  **END FUNCTION** |
| --- |

Figure A-2. Pseudo-code for $f_{d\_RMS}$.

| **FUNCTION** $\boldsymbol{f}_{\boldsymbol{d\_SIGMA}}$(*signal*)  $f_{spin}$: 11-16 Hz band  $f_{l}$: 4‑10 Hz band  $f_{h}$: 20-40 Hz band  $f_{\alpha}$: 7.5-10 Hz band  *windows* 🡨 Separate *signal* in windows of 4.2 s with 0.2 s overlap  $f_{d}$ 🡨 []  **FOR** window **IN** windows **DO**  Compute $\boldsymbol{ST}(t, f, window)$ over the 4-40 Hz band  $max\left( t \right)$ 🡨$\max_{f_{spin}} \left( \boldsymbol{ST}(t, f_{spin}) \right)$  $m_{l} \left( t \right)$ 🡨$\boldsymbol{mean}(\boldsymbol{ST}(t, f_{l}))$  $m_{h}\left( t \right)$ 🡨$\boldsymbol{mean}(\boldsymbol{ST}(t, f_{h}))$    $f_{d\_partial}=\left\{ \begin{matrix} 0 & \underset{f_{\alpha}}{\boldsymbol{IF} \boldsymbol{max}} \left( \boldsymbol{ST}\left( t, f_{\alpha} \right) \right)>max\left( t \right) \\ \frac{2*max\left( t \right)}{m_{l}\left( t \right)+m_{h}\left( t \right)} & \boldsymbol{ELSE} \end{matrix} \right.$  $f_{d}$.**append**($f_{d\_partial}$ over the 0.1-4.1 s range)  **RETURN** $f_{d}$  **END FUNCTION** |
| --- |

Figure A-3. Pseudo-code for $f_{d\_SIGMA}$.

| **FUNCTION** $\boldsymbol{f}_{\boldsymbol{d\_RSP}}$(*signal*)  *windows* 🡨 Separate *signal* in windows of 4.2 s with 0.2 s overlap  $f_{d}$ 🡨 []  **FOR** window **IN** windows **DO**  Compute $\boldsymbol{ST}(t, f, window)$ over the 0.5-40 Hz band  $f_{d\_partial}=\frac{\int_{11}^{16} \boldsymbol{ST}(t, f)df}{\int_{0.5}^{40} \boldsymbol{ST}(t, f)df}$  $f_{d}$.**append**($f_{d\_partial}$ over the 0.1-4.1 s range)  **RETURN** $f_{d}$  **END FUNCTION** |
| --- |

Figure A-4. Pseudo-code for $f_{d\_RSP}$.

| **FUNCTION** $\boldsymbol{f}_{\boldsymbol{d\_TEAGER}}$(*signal*)  $h$ 🡨 $\boldsymbol{filter}(signal)$  **RETURN** $h^{2}\left( n \right)-h\left( n-1 \right)h\left( n+1 \right)$  **END FUNCTION** |
| --- |

Figure A-5. Pseudo-code for $f_{d\_TEAGER}$.
